# Novel oxadiazole derivatives as potent inhibitors of α-amylase and α-glucosidase enzymes: Synthesis, *in vitro* evaluation, and molecular docking studies

**DOI:** 10.22038/IJBMS.2021.58429.12977

**Published:** 2021-12

**Authors:** Asma Bukhari, Humaira Nadeem, Muhammad Imran, Syed Aun Muhammad

**Affiliations:** 1 Riphah Institute of Pharmaceutical Sciences, Riphah International University Islamabad, 44000, Pakistan; 2 Department of Pharmacy, Iqra University H-9 Campus Islamabad, 44000, Pakistan; 3 Department of Biotechnology, Bahauddin Zakariya University (BZU), Multan, Pakistan

**Keywords:** α-Amylase enzyme, α-Glucosidase enzyme, Molecular docking, MTT assay, Oxadiazole

## Abstract

**Objective(s):**

Alpha-amylase and alpha-glucosidase enzyme inhibition is an effective and rational approach for controlling postprandial hyperglycemia in type II diabetes mellitus (DM). Several inhibitors of this therapeutic class are in clinical use but are facing challenges of safety, efficacy, and potency. Keeping in view the importance of these therapeutic inhibitors, in this study we are reporting 10 new oxadiazole analogs 5 (a-g) & 4a (a-c) as antidiabetic agents.

**Materials and Methods:**

The newly synthesized derivatives 5 (a-g) & 4a (a-c) were characterized using different spectroscopic techniques including FTIR,^1^HNMR, ^13^CNMR, and elemental analysis data. All compounds were screened for their *in vitro* α-amylase and α-glucosidase enzyme inhibitory potential, while two selected compounds (5a and 5g) were screened for cytotoxicity using MTT assay.

**Results:**

Two analogues 5a and 4a (a) exhibited strong inhibitory potential against α-glucosidase enzyme, i.e., IC_50_ value=12.27±0.41 µg/ml and 15.45±0.20 µg/ml, respectively in comparison with standard drug miglitol (IC_50_ value=11.47±0.02 µg/ml) whereas, one compound 5g demonstrated outstanding inhibitory potential (IC_50_ value=13.09±0.06 µg/ml) against α-amylase enzyme in comparison with standard drug acarbose (IC_50_ value=12.20±0.78 µg/ml). The molecular interactions of these active compounds in the enzymes’ active sites were evaluated following molecular docking studies.

**Conclusion:**

Our results suggested that these new oxadiazole derivatives (5a, 5g & 4a (a)) may act as promising drug candidates for the development of new alpha-amylase and alpha-glucosidase inhibitors. Therefore, we further recommend *in vitro* and *in vivo* pharmacological evaluations and safety assessments.

## Introduction

Diabetes mellitus (DM) is a metabolic disorder of multiple etiologies characterized by chronic hyperglycemia and abnormalities in carbohydrate, fat, and protein metabolism which result from irregularities in insulin secretion, insulin action, or both. The long-term effects of DM include retinopathy, neuropathy, amputation, and Charcot joints. Diabetic patients are also at higher risk of developing peripheral, cardiovascular, and cerebrovascular diseases (1). Polyuria, polydipsia, weight loss, polyphagia, impairment of growth, and susceptibility to certain infections may also accompany chronic hyperglycemia (2). Broadly, DM has been classified into two categories, type I: insulin-dependent diabetes mellitus (IDDM), the cause is an absolute deficiency of insulin secretion, and type II: non-insulin-dependent diabetes mellitus (NIDDM), the cause is a combination of resistance to insulin action and an insufficient compensatory insulin secretory response (3, 4). Type-II DM typically leads to metabolic syndrome, which also includes abdominal obesity, hypertension, hyperlipidemia, and increased coagulability (5). DM is a critical and expanding global health burden and estimates of prevalence are significant for relevant allocation of resources and monitoring of trends. Radical changes in work patterns, lack of physical activity, improved transportation, use of junk foods and prominent modifications in lifestyle over the past few decades have increased the incidence of diabetes worldwide. According to a report by WHO, the reported diabetic patients in the developing world were 84 million in 1995 and it will increase up to 228 million by 2025 (6). As DM is a long-term disease and requires long-term management, it needs time to explore new, effective, and safer agents (7). Some of the known management strategies include the use of sulfonylureas, biguanides, and thiazolidinediones (8, 9). Another effective approach to decrease postprandial hyperglycemia is to provide reduced meal-derived glucose absorption by inhibition of carbohydrate hydrolyzing enzymes, such as α-glucosidase and α-amylase, in the digestive organs (10-13). In the first step, the pancreatic amylase enzymes catalyze the digestion of starch into smaller oligosaccharides while in the next step; it is further processed into smaller absorbable glucose units with the action of α-glucosidases. This metabolic cycle ultimately increases the postprandial hyperglycemia, while its supervision may provide a therapeutic opportunity in controlling DM (14). DM can be successfully cured by α-glucosidase inhibitors, which have the ability to delay and reduce postprandial blood glucose levels (15-16). α-Glucosidase is involved in carbohydrate metabolism and has an important function in diabetes, cancer, and viral infections. α-Glucosidase has various biological activities and is considered an attractive drug target. At present, a number of α-glucosidase inhibitors have been discovered and studied. Voglibose, acarbose, and miglitol are the clinically used anti-diabetic agents which inhibit α-glucosidase competitively in the brush border of the small intestine and therefore delay the hydrolysis of carbohydrates, reducing postprandial hyperglycemia. However, continuous administration of these drugs may cause several side effects such as abdominal pain and diarrhea (17). Therefore, developing new α-glucosidase inhibitors lacking these problems is necessary.

**Figure 1:**
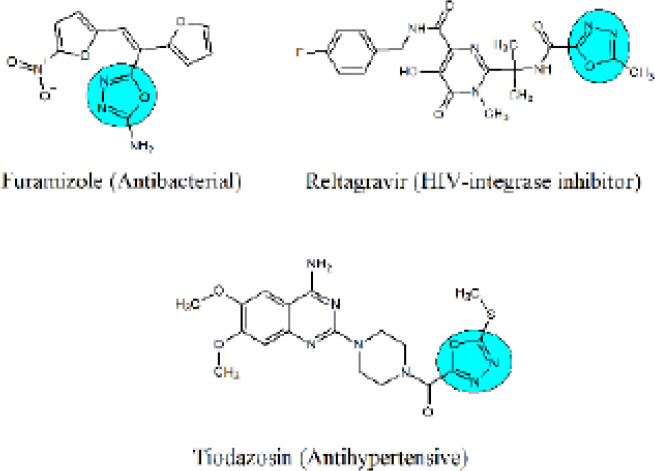
Commercially available drugs with the 1,3,4-oxadiazole nucleus

Oxadiazole is a five-membered heterocycle compound containing two carbon, two nitrogen, and one oxygen atom in the ring (18). Depending upon the position of the nitrogen atom, the oxadiazole ring may yield four different isomers (19). Among all these four isomeric forms,1,3,4-oxadiazole is widely investigated because of its significant interacting potential with various binding sites in the biological systems (20). Due to such versatility, medicinal chemists extensively used this pharmacophore in the design and synthesis of various therapeutic ligands. 1,3,4-oxadiazole derivatives have been reported to possess various pharmacological activities such as muscle relaxants, antimitotic, anticancer, antidiabetic, antimicrobial, antihypertensive, anticonvulsant, and anti-inflammatory agents. Similarly, 1,3,4-oxadiazoles analogues represent a unique metabolic profile and hydrogen bonding ability, which further signifies its medicinal importance (21-25). Some of the commercially available clinical drugs containing 1,3,4-oxadiazole nucleus are mentioned in Figure 1. 

The sulfur atom-containing compounds and respective derivatives have been implicated as important structural motifs in medicinal chemistry due to their biologically active nature (26, 27). Moreover, the versatile nature of Sulphur containing compounds has enabled it to become a part of many natural and synthetic drugs (28). Based upon this detailed insight, we synthesized 10 new 2-thione-1,3,4-oxadiazole derivatives as potential anti-diabetic agents. The newly synthesized compounds were screened for *in vitro *alpha-amylase and *in vitro *alpha-glucosidase inhibitory potential. Further, these compounds were screened for their binding potentials in the respective protein (alpha-amylase and alpha-glucosidase) pockets through molecular docking. Our results suggested that these compounds may provide a rational approach for the development of new and potent inhibitors of these diabetogenic enzymes.

## Materials and Methods

***Materials***

All used chemicals and solvents used were procured from Sigma-Aldrich (Germany), Merck (Germany), and Alfa Aesar (USA). Characterization of all synthesized compounds was performed using different spectroscopic techniques, FTIR (alpha Bruker, eco ATR),^1^HNMR, and ^13^CNMR spectra (Bruker AM-300-Spectrophotometer, tetramethylsilane (TMS) was used as internal standard). Deuterated solvents (Chloroform and DMSO) were used for recording the spectra while chemical shifts were presented as delta values with respect to TMS. Melting points were recorded using the digital Gallen kemp melting point apparatus. The synthesized final compounds were recrystallized using a suitable solvent. The progress of reactions was monitored using thin-layer chromatography (TLC). Solvent system (methanol: chloroform 1:9) and silica gel-60 HF_254_ plates were used. Phosphate buffer, starch, and di-nitro salicylic acid, Na_2_CO_3, _bovine serum albumin, p-nitrophenyl α-d-glucopyranoside, and α-glucosidase were used for anti-diabetic assays. 3-(4,5-dimethylthiazol-2-yl)-2,5-diphenyltetrazolium bromide, Dulbecco’s modified eagle’s medium (DMEM), fetal bovine serum, penicillin, and streptomycin were used for MTT assay. 

***Synthesis***

*General procedure for synthesis of 2-thion-1, 3, 4-oxadizole derivatives 5(a-g) and 4a(a-c)*

For preparation of 2-thione-1,3,4-oxadiazole derivatives 5(a-g) and 4a (a-c), two different schemes (I & II) were utilized. 5(a-g) derivatives were prepared following a four-step process (scheme I) whereas 4a(a-c) were synthesized following a three-step process (scheme II). In the first step of scheme I, substituted acids 1(a-g) were converted into their respective esters 2(a-g) using ethanol and acidic catalyst. Esters were converted into hydrazides 3(a-g), which were further used to synthesize 1,3,4-oxadiazole 4(a-g). In the final step, 2-thione-1,3,4-oxadiazole derivatives 5(a-g) were produced. In scheme II, pyridine rings containing substituted acids 1a(a-c) were utilized to prepare hydrazides 2a(a-c). In the second step, 1,3,4-oxadiazole 3a(a-c) were subsequently prepared following its reaction with 2-bromo ethanol to yield final products 4a(a-c).

*General procedure for synthesis of esters 2(a-g)*

For synthesis of esters 2(a-g), the already reported method was used with little modifications (29). Substituted acids 1(a-g) (0.032 mol) were refluxed in absolute ethanol (20 ml). To this mixture, 1 ml of concentrated sulphuric acid was added and the reaction continued until the completion. The reaction progress was monitored through TLC. After completion of the reaction, the solution was cooled, poured into ice water, and extracted with ethyl acetate. The organic layer was washed with water and 10% NaHCO_3_ solution, dried over anhydrous sodium sulfate, and evaporated to dryness to afford final esters.

*General procedure for preparation of hydrazides 3(a-g) and 2a(a-c)*

Respective esters 2(a-g) and substituted acids 1a(a-c) were dissolved in absolute methanol (50 ml) and added with hydrazine monohydrate (15 ml). The mixture was magnetically stirred and heated under reflux for 10-12 hr. The progress of the reaction was monitored through TLC (silica; chloroform: methanol 6:1). The excess ethanol and hydrazine were evaporated under reduced pressure that yielded corresponding hydrazides (30).

*General procedure for preparation of 1,3,4-oxadiazoles4(a-g) and 3a(a-c)*

A previously reported method was used for preparation of the 1,3,4-oxadiazole nucleus with little modifications (31). Already prepared hydrazides 3(a-g) and 2a(a-c) (0.0139, mol) were dissolved in the absolute ethanol (30 ml). To this solution, carbon disulfide (1.066 ml) was added, following the addition of potassium hydroxide (0.0139 mol dissolved in water 20 ml). This mixture was shaken vigorously and refluxed till complete evaporation of hydrogen disulfide. The reaction progress was monitored through TLC (ethyl acetate: petroleum ether 1:9). After completion of the reaction, the solution was concentrated to a small volume and the residue was dissolved in water. The solution was acidified (pH 2-3, dilute HCl) till the solid precipitates appeared, filtered, and recrystallized using suitable solvent (aq. ethanol). 

*Procedure for synthesis of 2-thion 1, 3, 4-oxadizolederivatives 5(a-g) and 4a(a-c)*

For preparation of final products 5(a-g) and 4a(a-c), a reaction mixture was prepared using equimolar ratios of respective oxadiazoles (0.0056, mol), 2-bromo ethanol(0.398 ml) and potassium carbonate (0.0056, mol) were dissolved in ethanol (10 ml) and water (10 ml). The reaction mixture was magnetically stirred at room temperature till the solid precipitates appeared. The completion of the reaction was assessed through TLC (chloroform: methanol 9:1). After completion of the reaction, the compound was air-dried and recrystallized using a suitable solvent (absolute ethanol).

***Spectral analysis of 5(a-g) and 4a(a-c)***

*2-{5-[(2-hydroxyethyl) sulfanyl]-1, 3, 4-oxadiazol-2-yl} phenol (5a)*

Orange solid; Yield 80 %; m.p. 165–170 ^°^C; R_f_ value= 0.64 (methanol :chloroform 1:9); FTIR (cm^-1^); 1629 (C=C), 1636 (C=N), 1127 (C-O), 3241 (O-H); ^1^HNMR (δ ppm, DMSO): 3.38(t, 2H, -CH_2_), 3.54(t, 2H, -CH_2_), 7.49-7.91 (m, 4H, J=8.1Hz, Aryl H).^13^C-NMR (DMSO-d6, δ ppm); (31.1, 1C), (60.8, 1C), (112.6, 1C), (117.3, 1C), (119.9, 1C), (127.3, 1C), (131.9, 1C), (158.1, 1C), (164.6, 1C), (165.3, 1C).Elemental analysis for C_10_H_10_O_3_N_2_S: C, 49.91%; H, 4.24%; O, 20.15%; N, 11.78%. Found: C, 49.89%; H, 4.29%; O, 20.09%; N, 11.79%.

*4-{5-[(2-hydroxyethyl) sulfanyl]-1, 3, 4-oxadiazol-2-yl} phenol (5b)*

Orange solid; Yield 70 %; m.p. 195 ^°^C; R_f _value=0.64 (methanol : chloroform 1:9); FTIR (cm^-1^); 1604(C=C), 1636 (C=N), 1180 (C-O), 3345 (O-H); ^1^HNMR (δ ppm, DMSO): 3.36(t, 2H, -CH_2_), 3.56(t, 2H, -CH_2_), 7.36(d, 2H, J= 8.1Hz, Aryl H), 7.79(d, 2H, J= 8.2Hz, Aryl H).^ 13^C-NMR (DMSO-d6, δ ppm); (30.9, 1C), (61.4, 1C), (115.0, 2C), (122.8, 1C), (128.9, 2C), (157.8, 1C), (164.1, 1C), (165.5, 1C). Elemental analysis for C_10_H_10_O_3_N_2_S: C, 50.01%; H, 4.24%; O, 20.15%; N, 11.78%. Found: C, 50.11%; H, 4.27%; O, 20.17%; N, 11.80%.

*2-{[5-(2-chlorophenyl)-1, 3, 4-oxadiazol-2-yl] sulfanyl} ethan-1-ol (5c)*

Orange solid; Yield 75 %; m.p. 165 °C; R_f_ value=0.63 (methanol:chloroform 1:9); FTIR (cm^-1^); 1650 (C=C), 1625 (C=N), 1150 (C-O), 3301 (O-H); ^1^HNMR (δ ppm, DMSO): 3.38(t, 2H, -CH_2_), 3.55(t, 2H, -CH_2_), 7.26-7.99(m, 4H, J=7.9Hz, Aryl H).^13^C-NMR (DMSO-d6, δ ppm); (31.4, 1C), (60.6, 1C), (126.4, 1C), (128.0, 1C), (130.4, 1C), (130.8, 1C), (131.1, 1C), (133.2, 1C), (164.9, 1C), (166.1, 1C). Elemental analysis for C_10_H_9_N_2_O_2_ClS: C, 47.14%; H, 3.63%; O, 12.47%; N, 10.89%. Found: C, 47.09%; H, 3.67%; O, 12.41%; N, 10.80%.

*2-{[5-(3-bromophenyl)-1, 3, 4-oxadiazol-2-yl] sulfanyl} ethan-1-ol (5d)*

Orange solid; Yield 60 %; m.p. 186–190 ^°^C; R_f_ value=0.62 (methanol : chloroform 1:9); FTIR (cm^-1^); 1592 (C=C), 1629 (C=N), 1123 (C-O), 3255 (O-H); ^1^HNMR (δ ppm, DMSO): 3.37(t, 2H, -CH_2_), 3.54(t, 2H, -CH_2_), 7.23-7.75 (m, 4H, J=7.9Hz, Aryl H).^13^C-NMR (DMSO-d6, δ ppm); (31.1, 1C), (60.8, 1C), (118.2, 1C), (125.5, 1C), (127.1, 1C), (129.9, 1C), (130.7, 1C), (133.0, 1C), (163.8, 1C), (165.1, 1C). Elemental analysis for C_10_H_9_O_2_N_2_BrS: C, 40.01%; H, 3.10%;O, 9.91%; N, 9.41%. Found: C, 40.03%; H, 3.07%; O, 9.95%; N, 9.35%.

*2-{[5-(3-iodophenyl)-1, 3, 4-oxadiazol-2-yl] sulfanyl} ethan-1-ol (5e)*

Orange solid; Yield 70 %; m.p. 170 °C; R_f_ value=0.62 (methanol : chloroform 1:9); FTIR (cm^-1^); 1599 (C=C), 1634 (C=N), 1131 (C-O), 3240 (O-H); ^1^HNMR (δ ppm, DMSO): 3.34(t, 2H, -CH_2_), 3.43(t, 2H, -CH_2_), 7.15-7.67 (m, 4H, J=7.8Hz, Aryl H). ^13^C-NMR (DMSO-d6, δ ppm); (32.1, 1C), (62.2, 1C), (91.6, 1C), (125.3, 1C), (127.1, 1C), (128.5, 1C), (137.9, 1C), (138.5, 1C), (163.8, 1C), (164.8, 1C). Elemental analysis for C_10_H_9_IN_2_O_2_S: C, 34.50%; H, 2.70%; N, 8.05%; O, 9.19%. Found: C, 34.53%; H, 2.63%; N, 8.02%; O, 9.17%.

*5-{5-[(2-hydroxyethyl) sulfanyl]-1, 3, 4-oxadiazol-2-yl} benzene-1,2,3-triol (5f)*

Orange solid; Yield 72 %; m.p. 265–270 ^°^C; R_f_ value=0.63 (methanol : chloroform 1:9); FTIR (cm^-1^); 1650 (C=C), 1622 (C=N), 1041 (C-O), 3375 (O-H);^1^HNMR (δ ppm, DMSO): 3.46(t, 2H, -CH_2_), 3.56(t, 2H, -CH_2_), 7.52 (d, 2H, J=2.6Hz, Aryl H).^13^C-NMR (DMSO-d6, δ ppm); (31.1, 1C), (60.7, 1C), (111.1, 2C), (125.7, 1C), (137.6, 1C), (146.9, 2C), (164.5, 1C), (165.1, 1C). Elemental value for C_10_H_10_N_2_O_5_S: C, 44.44%; H, 3.73%; N, 10.37%; O, 29.60%. Found: C, 44.40%; H, 3.77%; N, 10.39%; O, 29.64%.

*2-[(5-phenyl-1, 3, 4-oxadiazol-2-yl) sulfanyl] ethan-1-ol (5g)*

Orange solid; Yield 80 %; m.p. 207–210 ^°^C; R_f _value=0.63 (methanol : chloroform 1:9); FTIR (cm^-1^); 1635 (C=C), 1633 (C=N), 1140 (C-O), 3257 (O-H); ^1^HNMR (δ ppm, CHCl_3_): 3.42(t, 2H, -CH_2_), 3.26(t, 2H, -CH_2_), 7.47-7.96 (m, 5H, J= 8.1Hz, Aryl H).^13^C-NMR (DMSO-d6, δ ppm); (31.3, 1C), (62.0, 1C), (122.9, 1C), (127.0, 2C), (129.0, 3C), (164.6, 1C), (165.3, 1C). Elemental analysis for C_10_H_10_O_2_N_2_S: C, 53.90%; H, 4.61%; O, 14.40%; N, 12.59%. Found: C, 53.97%; H, 4.57%; O, 14.43%; N, 12.63%.

*2-{[5-(pyridin-2-yl)-1, 3, 4-oxadiazol-2-yl] sulfanyl} ethan-1-ol 4a(a)*

Orange solid; Yield 35 %; m.p. 145 ^°^C; R_f _value=0.63 (methanol:chloroform 1:9); FTIR (cm^-1^); 1598 (C=C), 1631 (C=N), 1049 (C-O), 3360 (O-H); ^1^HNMR (δ ppm, DMSO): 3.38(t, 2H, -CH_2_), 3.53(t, 2H, -CH_2_), 7.61-7.93 (m, 4H, J=8.1Hz, Aryl H).^13^C-NMR (DMSO-d6, δ ppm); (30.9, 1C), (60.8, 1C), (123.4, 1C), (124.1, 1C), (136.8, 1C), (145.7, 1C), (149.7, 1C), (164.3, 1C), (165.5, 1C). Elemental analysis for C_9_H_9_N_3_O_2_S: C, 48.42%; H, 4.06%; N, 18.82%; O, 14.33%. Found: C, 48.46%; H, 4.08%; N, 18.80%; O, 14.31%.

*2-{[5-(pyridin-3-yl)-1, 3, 4-oxadiazol-2-yl] sulfanyl} ethan-1-ol 4a(b)*

Orange solid; Yield 80 %; m.p. 185 ^°^C; R_f_ value=0.63 (methanol:chloroform 1:9); FTIR (cm^-1^); 1590 (C=C), 1630 (C=N), 1060 (C-O), 3380 (O-H); ^1^HNMR (δ ppm, DMSO): 3.36(t, 2H, -CH_2_), 3.56(t, 2H, -CH_2_), 7.42-8.01 (m, 4H, J= 8.2Hz, Aryl H). ^13^C-NMR (DMSO-d6, δ ppm); (31.1, 1C), (61.8, 1C), (123.6, 1C), (123.9, 1C), (134.5, 1C), (148.5, 1C), (149.6, 1C), (163.3, 1C), (165.6, 1C). Elemental analysis for C_9_H_9_N_3_O_2_S: C, 48.42%; H, 4.06%; N, 19.02%; O, 14.33%. Found: C, 48.46%; H, 4.07%; N, 19.10%; O, 14.31%.

*2-{[5-(pyridin-4-yl)-1,3,4-oxadiazol-2-yl] sulfanyl} ethan-1-ol 4a(c)*

Orange solid; Yield 60 %; m.p. 180 °C; R_f_ value=0.62 (methanol:chloroform 1:9); FTIR (cm^-1^); 1635 (C=C), 1627 (C=N), 1185 (C-O), 3241 (O-H); ^1^HNMR (δ ppm, DMSO):3.35(t, 2H, -CH_2_), 3.80(t, 2H, -CH_2_), 7.79(d, 2H, J= 5.1Hz, Aryl H), 8.27(d, 2H, J= 6.5Hz, Aryl H).^13^C-NMR (DMSO-d6, δ ppm); (31.1, 1C), (60.8, 1C), (119.6, 2C), (125.6, 1C), (150.6, 2C), (164.6, 1C), (165.3, 1C). Elemental analysis for C_9_H_9_N_3_O_2_S: C, 48.42%; H, 4.06%; N, 19.03%; O, 14.33%. Found: C, 48.46%; H, 4.08%; N, 19.06%; O, 14.34%.

***Antidiabetic activity***

*In vitro alpha-amylase inhibitory activity*

The α-amylase inhibitory activity of the newly synthesized compounds 5(a-g) and 4a(a-c) was determined according to a previously described method (32, 33). 500 μl of test compounds (1–100 μg/ml) were incubated with 500 μl of porcine pancreatic amylase (0.5 mg/ml in 100 mM phosphate buffer, pH 6.8) for 15 min at 25 ^°^C. To this reaction mixture, 1% starch solution was added (500 μl in 0.2 mM phosphate buffer, pH 6.8) and incubated for an additional 10 min. 1 ml of DNS (di-nitro-salicylic-acid) color reagent was added to arrest the reaction. The reaction mixture was boiled for 10 min and then brought to ambient temperature. The absorbance of the resulting mixture was measured at 540 nm and the inhibitory activity was calculated using the following formula. Acarbose was used as a standard drug.

% Inhibition =(As_tandard_ -A_sample_)/A_standard_× 100

The IC_50_ was calculated using non-linear regression plot of % inhibition versus concentrations with the help of GraphPad Prism, version 6.0.

*In vitro alpha-glucosidase inhibitory activity*

The α-glucosidase inhibitory activity was determined according to a previously reported method with slight modifications(19). The α-glucosidase solution was prepared by dissolving 1 mg in 100 mL phosphate buffer (pH 6.8) comprising 200 mg bovine serum albumin. A 500 μl solution was prepared by adding 10 μl of test compounds (1-100 μg/ml) and 490 μl of phosphate buffer (pH 6.9). 250 μl of 5mM p-nitrophenyl α-d-glucopyranoside (p-NPG) were added to this reaction mixture and incubated for 10 min at 37 ^°^C. Furthermore, 250 μl of α-glucosidase (0.15 units/ml) were added to it and incubation continued for another 10 min at 37 ^°^C. Finally, 2000 μl of Na_2_CO_3_(200mM) were added to conclude this inhibition. The α-glucosidase inhibitory activity was calculated as a measure of p-nitrophenol released from p-NPG. The absorbance was measured at 400 nm, using miglitol as a standard drug. The percentage inhibition was calculated using the following formula;

% Inhibition = (^A^_standard_^- A^_sample_) / ^A^_standard_ × 100.

***MTT assay for cytotoxicity analysis***

Cytotoxic activity of compounds was evaluated in 96-well flat-bottomed microplates by using the standard MTT (3-[4, 5-dimethylthiazole-2-yl]-2, 5-diphenyl-tetrazolium bromide) colorimetric assay (34). For this purpose, 3T3 (mouse fibroblast, ATCC # CRL-1658, Lot # 59049195) cells were cultured in Dulbecco’s Modified Eagle Medium, supplemented with 5% fetal bovine serum (FBS), 100 IU/ml penicillin, and 100 µg/ml streptomycin in 75 cm^2^ flasks, and kept in a 5% CO_2_ incubator at 37 ^o^C. Exponentially growing cells were harvested, counted with a hemocytometer, and diluted with a particular medium. Cell culture with concentration of 5x104 cells/ml was prepared and introduced (100 µl/well) into 96-well plates. After overnight incubation, the medium was removed and 200 µl of fresh medium was added with various oxadiazole concentrations (30 µM, 15 µM, 7.5 µM, 3.75 µM, and 1.875 µM). After 48 hr, 200 µl MTT (0.5 mg/ml) was added to each well and incubated further for 4 hr. Subsequently, 100 µl of DMSO was added to each well. The extent of MTT reduction to formazan within cells was calculated by measuring the absorbance at 540 nm, using a microplate reader (Spectra Max Plus, Molecular Devices, CA, USA). The cytotoxicity was recorded as concentration causing 50% growth inhibition (IC50) for 3T3 cells. The percent inhibition was calculated by using the following formula;

% Inhibition=100-((mean of O.D of test compound-mean of O.D of negative control)/ (mean of O.D of positive control - mean of O.D of negative control) *100).

The results (% inhibition) were processed by using SoftMax Pro software (Molecular Device, USA).

***Molecular docking***

Docking analysis was performed against alpha-amylase enzyme (PDB code 3DHP) and alpha-glucosidase enzyme (PDB code 3WY1) to compare the relative affinity of the selected ligands (5a, 4a(a), and 5g) in the protein pockets. The protein structure was downloaded from RCSB Protein Data Bank Site(35-37). 3-D optimization of the ligand structures was done and saved in mol format. ArgusLab was used to convert ligands from mol format to pdb format (38). For Grid parameters, Auto Dock tools were used to provide the search space coordinates to Auto Dock Vina for docking of ligand molecules to α-amylase and α-glucosidase protein structures (39). The grid parametric values for α-amylase were adjusted to x=11, y=39, and z=17 with spacing of 1.0 Å and for α-glucosidase amylase they were adjusted to x=-11, y=-18, and z=0 with spacing of 1.0 Å. Binding energies (Kcal/mol) and best binding conformations were obtained as an output. Discovery Studio was used to visualize the binding conformations with the lowest energy coefficients. The amino acids involved in the ligand-protein interactions were analyzed using the spatial (3D) and linear (2D) interaction maps.

## Results

***Chemistry***

Ten new 1,3,4-oxadiazole derivatives were synthesized in our lab following the schemes (I&II). In scheme I, a four-step process was utilized to yield 2-thione-1,3,4-oxadiazoles while in scheme II, a three-step process was followed to synthesize final products. The purity of synthesized compounds was confirmed with the help of thin-layer chromatography (TLC). The detailed structure elucidation of the final compounds 5(a-g) and 4a(a-c) was performed using FTIR,^1^HNMR, ^13^CNMR, and elemental analysis. 

***In vitro Alpha-amylase inhibitory activity***

The newly synthesized compounds 5(a-g) & 4a(a-c) were screened following alpha-amylase inhibitory assay and IC_50_ value (µg/ml±SEM) was calculated (Table 1). The alpha-amylase inhibitors were found to prevent or slow down the absorption of dietary starch by hindering the hydrolysis of 1,4 glycosidic linkages of starch and other oligosaccharides into disaccharides which are further converted to glucose (40). The obtained results showed diversity depending upon the functional groups attached. Compound 5g (aryl derivative) exhibited strong inhibitory activity with 92.16 % inhibition and IC_50_ value of 13.09±0.06 µg/ml. Similarly, the IC_50_ values of other compounds 5(a-f), 4a(a-c) were calculated ranging from 90.02±0.08 µg/ml to 394.34±0.63 µg/ml. Whereas, the standard drug acarbose showed 92.47 percent of inhibition with an IC_50_ value of 12.20±0.78 µg/ml. The results demonstrated that the hydroxyl group in compounds 5a and 5b showed moderate IC_50_ results, i.e., 60.02±0.08 µg/ml and 64.8±1.07 µg/ml, respectively. Similarly, 5c, 5d, and 5e also inhibited the α-amylase enzyme but to a lesser extent, maybe due to mild electron-donating effects of attached electronegative elements (41). The compound 5g showed significant α-amylase inhibitory potential, almost similar to the positive control (acarbose).

***In vitro alpha-glucosidase inhibitory activity***

All synthesized compounds 5(a-g) and 4a(a-c) were evaluated for potential alpha-glucosidase inhibitory activity. The percent inhibitions and their corresponding IC_50_ values were mentioned in Table 2. The hydroxylated compound (5a) exhibited strong inhibitory activity (98.14%) having an IC_50_ value of 12.27±0.41 µg/ml in comparison with other compounds of the same series 5 (a-g). These results were quite similar to that of standard positive control miglitol (98.9%, IC_50_ value=11.47±0.02 µg/ml). 

Similarly, the other compounds of this series containing electronegative moieties (5b, 5c, 5d) also demonstrated good inhibitory potential but less than their hydroxyl moiety-containing counterpart as consistent with the previous studies (19). The induction of the pyridine ring showed different activities subjected to the position of ring nitrogen. Pyridine-2′ -yl derivative, as in compound 4a (a-c) showed potent inhibitory effects (97.20 %, IC_50_ value=15.45±0.20). This activity was decreased to many folds, when it was replaced with pyridine-4′ -yl, as in compound 4a (c) (16.3 %, IC_50_ value=299.11±0.31). Pyridine-3′ -yl derivative 4a (c) was found to be unsuitable to interact with the enzyme as it showed no activity at all. These results were similar to the previous reports (42).

***In vitro cytotoxicity assay***

**Figure 2:**
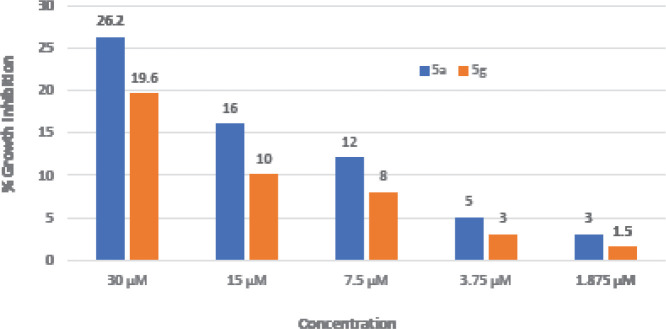
*In vitro* cytotoxicity analysis of compounds 5a and 5g at varying concentrations (concentration vs % growth inhibition)

Cytotoxic activity of compounds was evaluated in 96-well flat-bottomed microplates by using the standard MTT colorimetric assay. The results of the cytotoxicity assay of compounds 5a and 5b have been presented as percent inhibition in Figure 2. It can be observed that the cytotoxic effect was there but not significant enough. Compound 5a showed 26.2 % inhibition while test compound 5g showed 19.6 % inhibition at 30 µM concentration. The cytotoxic effect of selected oxadiazole derivative 5a was slightly more than the other derivative 5g in the same concentration ranges (Figure 2).

***Molecular docking***

The binding energy values between ligand and target proteins after docking have been mentioned in Table 3. While the distances of protein-ligand interactions are mentioned in Table 4. The results revealed that all ligands (5a, 5g, and 4a (a)) were well accommodated in the protein pockets of alpha-amylase and alpha-glucosidase.

**Figure 3:**
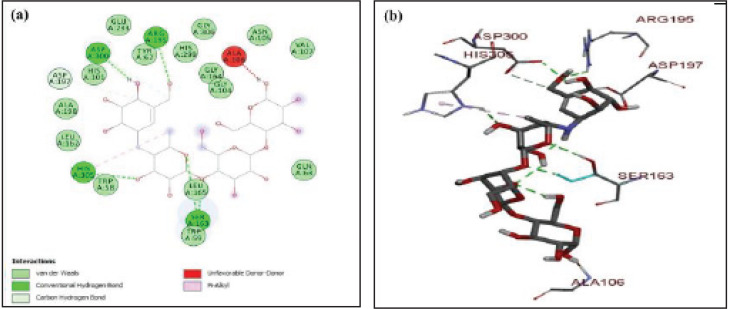
(a-b) Docked poses of positive control (standard drug) acarbose in the protein pocket of α-amylase; PDB id: 3dhp; a) 2D interactions of acarbose with surrounding amino acids of alpha-amylase; b) 3D interactive acarbose with amino acids of protein alpha-amylase

The molecular docking analysis of standard drug acarbose with the protein pocket of alpha-amylase is demonstrated in Figure 3 (a-b). The stable conformation of acarbose and α-amylase complex demonstrated the lowest binding energy of ^_^8.1 kcal/mol. Acarbose was stabilized in the protein pocket of alpha-amylase involving many conventional hydrogen bonding and van der Waals forces. Four different hydrogen bonds were observed between hydroxyl functional groups of acarbose and amino acids ARG:195, ASP:300, HIS A:305, and SER A:163, respectively. The cyclohexene ring was stabilized using van der Waals forces by amino acid ASP A:197. Some of the amino acids were observed as involved in stabilizing the alkyl groups of the standard drug by generating pi-alkyl bonding.

**Figure 4:**
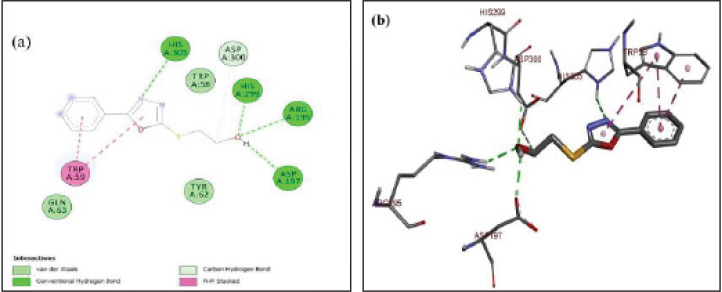
(a-b) Docked poses of 5g in the protein pocket of α-amylase; PDB id: 3dhp; a) 2D interactions of ligand (5 g) with surrounding amino acids of alpha-amylase; b) 3D interactive ligand (5 g) with amino acids of protein alpha-amylase

**Figure 5:**
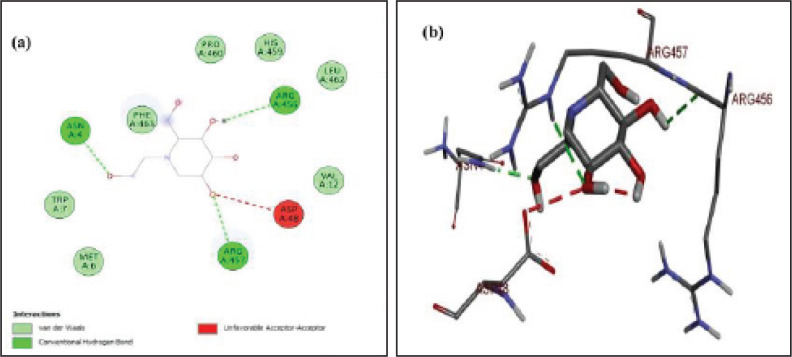
(a-b) Docked poses of positive control (standard drug) miglitol in the protein pocket of α-glucosidase; PDB id:3wy1; a) 2D interactions of miglitol with surrounding amino acids of alpha-glucosidase; b) 3D interactive miglitol with amino acids of protein alpha-glucosidase

The protein-ligand complex of 5g with α-amylase is shown in Figure 4 (a-b). The nitrogen of the oxadiazole ring showed conventional hydrogen bonding with HIS A:305. While the hydroxyl group attached to 5g further stabilized the protein-ligand complex by establishing strong hydrogen bonding with HIS A:299, ARG A:195, and ASP A:197. The aromatic rings of the ligand were stabilized by hydrophobic contact through Pi-Pi stacking with amino acid TRP A:59. The alkyl groups also developed van der Waals interactions with amino acid ASP A:300. The lowest binding energy of ligand-protein complex was ^_^6.6 kcal/mo. The docking analysis of the standard drug (miglitol) with alpha-glucosidase protein is presented in Figure 5 (a-b). In this ligand-protein complex, the amino acid residue ARG A:456 established conventional hydrogen bonding with the hydroxyl group of miglitol. While the other two strong hydrogen bondings were observed between hydroxyl group & ASN A:4 and –OH group & ARG A:457 to stabilize this complex. The lowest binding energy of this ligand-protein complex was ^_^8.8 kcal/mol. 

**Figure 6:**
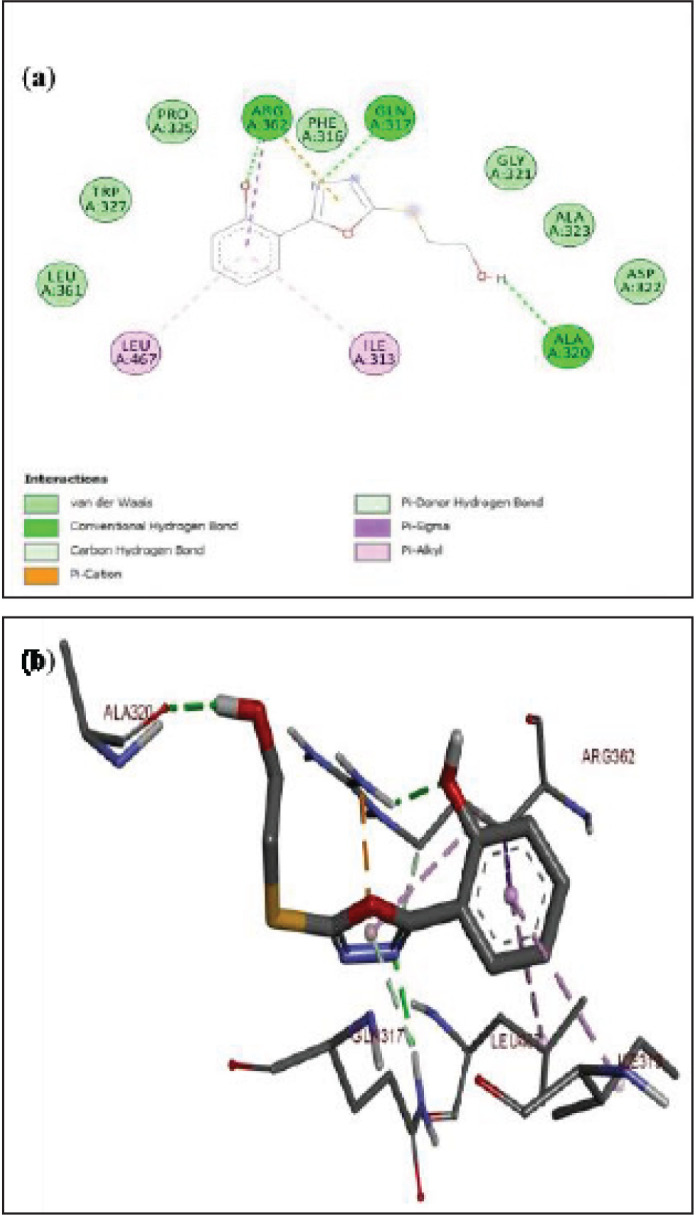
(a-b) Docked poses of ligand 5a in the protein pocket of α-glucosidase; PDB id:3wy1; a) 2D interactions of ligand (5a) with surrounding amino acids of alpha-glucosidase; b) 3D interactive ligand (5a) with amino acids of protein alpha-glucosidase

**Figure 7:**
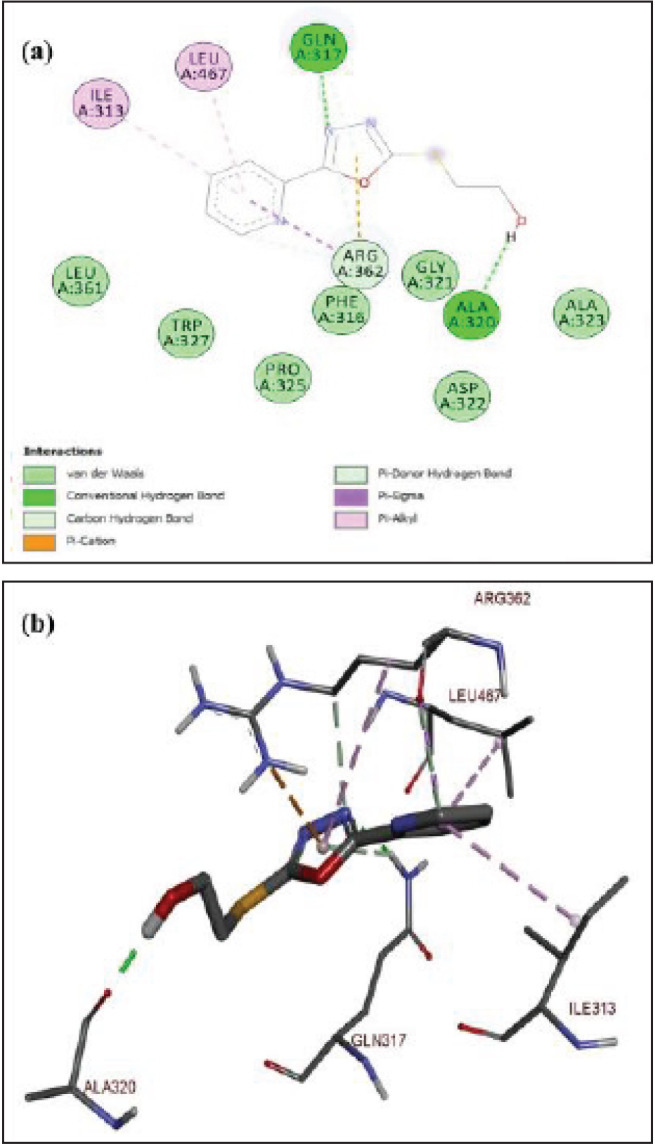
(a-b) Docked poses of ligand 4a (a) in the protein pocket of α-glucosidase; PDB id:3wy1; a) 2D interactions of ligand 4a(a) with surrounding amino acids of alpha-glucosidase; b) 3D interactive ligand 4a (a) with amino acids of protein alpha-glucosidase

The protein-ligand complex of α-glucosidase and 5a is shown in Figure 6 (a-b). The phenyl ring in ligand 5a was stabilized by π-sigma hydrophobic contact with amino acid residues LEU A:467 and ILE A:313. The ligand 5a was further stabilized by conventional hydrogen bonding between amino acid residue ALA A:320 and a hydroxyl group. Another hydrogen bond was established between the phenolic –OH and ARG A:362. The oxadiazole ring was stabilized in the complex through hydrophobic π-cation interactions. Whereas, the nitrogen of oxadiazole established a strong hydrogen bonding with amino acid residue GLN A:317. The lowest binding energy of this ligand-protein complex was ^_^9.7 kcal/mol. The strong interaction of this compound with the residues and potent activity may be due to the presence of the number and position of –OH groups (45). The docking analysis of compound 4a (a) and protein alpha-glucosidase is displayed in Figure 7 (a-b). The nitrogen atom of the oxadiazole ring established conventional hydrogen bonding with amino acid residue GLN A:317 to stabilize it. The phenyl ring of ligand 4a (a) showed hydrophobic interactions with amino acids LEU A:467 and ILE A:313. Whereas, the strong hydrogen bonding between the hydroxyl group and amino acid residue ALA A:320 further stabilized this ligand in the protein pocket of alpha-glucosidase. The lowest binding energy of this protein-ligand complex was ^_^6.5 kcal/mol. 

## Discussion

DM is a chronic disorder known for its associated complications that usually lead to cardiovascular and cerebrovascular disorders. Management of DM therefore demands intensive investigation. Scientists have investigated oxadiazoles and their derivatives for anti-cancer, anti-microbial, anti-diabetic, anti-hypertensive, and anticonvulsant activity. Oxadiazole derivatives have been found to possess good hydrogen-bonding ability and unique metabolic activity. In the present work, 10 new 2-thione-1,3,4-oxadiazole analogues 5(a-e) & 4a(a-c) were synthesized and investigated for potential anti-diabetic activities. All synthesized compounds were obtained as solids in good yields which were further re-crystallized using suitable solvents. The melting points of final products varied ranging from 145 ^°^C to 270 ^°^C. Some of the prominent functionalities were noticed in FTIR spectra of 5(a-g) and 4a (a-c). The stretching frequency for C=C (aromatic) was found in the range of 1590–1650 cm^-1^, C=N peak at 1622-1636 cm^-1^, C-O peak at 1041- 1185 cm^-1^, and OH stretch was witnessed at 3221-3380 cm^1^. Similarly, in ^1^H-NMR, a triplet of methylene protons, attached to the sulfur atom of the oxadiazole ring, appeared at 3.34-3.56 ppm in all the newly synthesized compounds. Another triplet of methylene proton attached to the OH group resonated slightly downfield in the range 3.2–3.8ppm. While the protons in the aromatic region appeared at the 7.15-8.01 ppm range with doublet and triplet signals. The hydroxyl proton was noticed in the range 4.0–7.0ppm. In a similar fashion, in ^13^CNMR, the methylene carbons appearing at 31.1 ppm and 60.8ppm confirmed the final formation of 2-thione-1,3,4-oxadiazole derivatives.

It has been previously reported in the literature that α-amylase inhibition is one of the effective methods to control diabetes (43). The α-amylase inhibitors delay the glucose absorption rate and regulate the serum blood glucose levels in hyperglycemic patients (29). One of the important attributes, designated with the attachment of oxadiazole moiety, is the improvement of the pharmacokinetic profile of the drug (44). Our results demonstrated that the hydroxyl group in compounds 5a and 5b showed moderate IC_50_ results, i.e., 60.02±0.08 µg/ml and 64.8±1.07 µg/ml, respectively. Similarly, 5c, 5d, and 5e also inhibited the α-amylase enzyme but to a lesser extent, maybe due to mild electron-donating effects of attached electronegative elements (41). The compound 5g showed significant α-amylase inhibitory potential (i.e., 92.16 % inhibition, IC_50_ value=13.09±0.06 µg/ml), almost similar to the positive control (acarbose). In this context, our synthesized compounds especially 5g may act as potential candidates for glycemic control in diabetic patients. 

Similarly, inhibition of α-glucosidase enzyme is an effective strategy for controlling postprandial hyperglycemia in diabetic patients (45). The activity of α-glucosidase inhibitors is dependent upon their effective binding to the carbohydrate-binding region of α-glucosidase enzymes. Whereas, the down-regulation of these enzymes slows down the digestion of carbohydrates, which subsequently quashes the rise of blood glucose levels (46). The hydroxylated compound (5a) exhibited strong inhibitory activity (98.14%) having an IC_50_ value of 12.27±0.41 µg/ml in comparison with other compounds of the same series 5(a-g). These results were quite similar to those of standard positive control miglitol (98.9%, IC_50_ value=11.47±0.02µg/ml). The *in vitro* cytotoxicity of compounds 5a and 5b was investigated at varying concentrations using an MTT assay. In the cell group where the maximum concentration of test compounds (30 µM) was used, the percent inhibition was found to be a mere 26.2% and 19.6% for 5a and 5g, respectively. On the other hand, in cell groups where the lowest concentration of oxadiazole analogs (1.875 µM) was employed, the cytotoxic effect was almost negligible (percent inhibition was 3% for 5a whereas for 5g it was about 1.5%.). From the MTT results, we can conclude that our newly synthesized 2-thione-1,3,4-oxadiazole analogs (5a & 5g) showed negligible cytotoxic effects against 3T3 (mouse fibroblast) cells.

Depending upon the strong inhibitory potential of 5g against α-amylase and of 5a & 4a(a) against α-glucosidase enzymes, the molecular docking studies were performed to further evaluate the molecular binding patterns of these ligands with the active sites of proteins (α-amylase & α-glucosidase). Molecular docking studies demonstrated that all ligands were found to be accommodated in the protein pockets of alpha-amylase and alpha-glucosidase. All docked ligands 5g, 5a, and 4a(a) showed a good binding affinity with the selected protein pockets, which correlate with the biological activities. 

**Scheme I:**
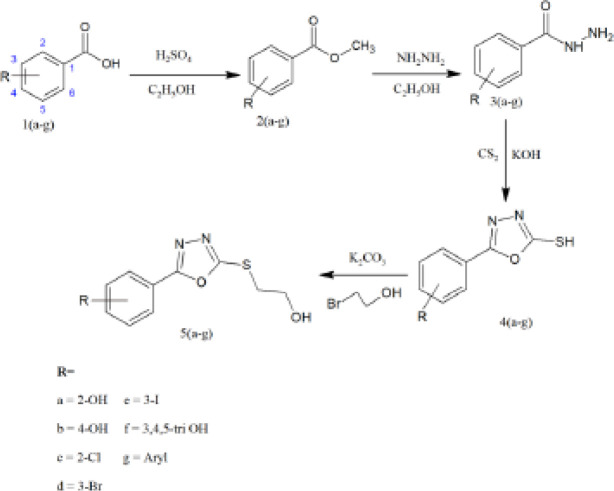
Synthesis of 2-substituted phenyl -1, 3, 4-oxadiazole-2-thion derivatives

**Scheme II:**
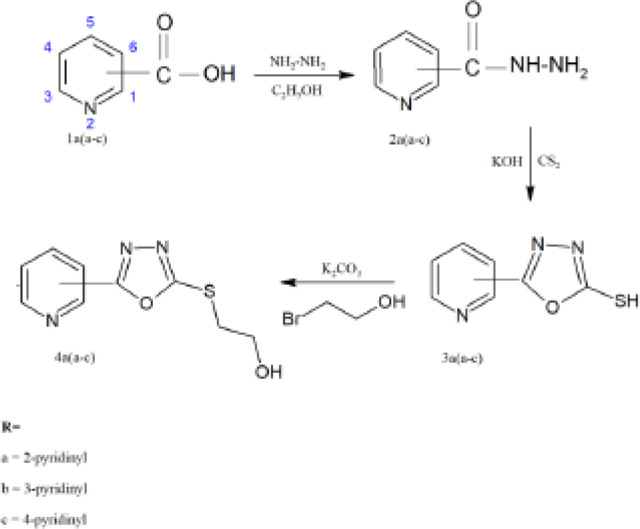
Synthesis of 5-pyridyl-1, 3, 4-oxadiazole-2-thion derivative

**Table 1:** Alpha-amylase inhibition activity of 2-thione-1,3,4-oxadiazole derivatives

Compound	IC_50_ µg/ml ± SEM	% Inhibition
5a	60.02 ± 0.08	56.41
5b	64.8 ± 1.07	50.11
5c	68.45 ± 0.15	49.41
5d	89.77 ± 0.91	43.6
5e	97.1 ± 0.80	40.2
5f	208.54 ± 0.14	19.04
5g	13.09 ± 0.06	92.16
4a(a)	291.12 ± 0.02	9.42
4a(b)	185.44 ± 0.36	26
4a(c)	394.34 ± 0.63	3.21
Acarbose	12.20± 0.78	92.47

SEM = Standard error mean

**Table 2:** Alpha-glucosidase inhibition activity of 2-thione-1,3,4-oxadiazole derivatives

Compound	IC_50_ µg/ml ± SEM	% Inhibition
5a	12.27 ± 0.41	98.14
5b	62.01 ± 0.92	41.8
5c	69.04 ± 0.27	40.04
5d	73.61 ± 0.02	39.7
5e	68.02 ± 0.43	40.2
5f	273.06 ± 0.04	9.04
5g	80.8 ± 0.52	36.16
4a(a)	15.45 ± 0.20	97.2
4a(b)	No effect	NA
4a(c)	299.11 ± 0.31	16.3
Miglitol	11.47 ± 0.02	98.9

SEM = Standard error mean

**Table 3:** Binding energy values of ligands with respective proteins after docking

**Protein Target**	**Ligand**	**Binding energy (kcal/mol)**
α-amylase	**5g**	-6.6
	**Acarbose**	-8.1
α-glucosidase	**5a**	-9.7
	**4a(a)**	-6.5
	**Miglitol**	-8.8

Acarbose and miglitol were used as standard drugs (positive control)

**Table 4:** Interacting distances between proteins and ligands

**Compound**	** Amino acids involved**	** Distance (Å)**
**5g**	TRP 59, GLN 63, TYR 62, ASP 197, ARG 195, HIS 299, TRP 58, ASP 300, HIS 305	5.19, 4.09, 4.73, 5.63, 5.45, 6.14, 4.60
**5a**	LEU 361, TRP 327, PRO 325, ARG 362, PHE 316, GLN 317, GLY 321, ALA 323, ASP 322, ALA 320, ILE 313, LEU 467	2.95, 3.78, 3.82, 4.75, 4.26, 3.87, 3.93, 4.21, 4.45
**4a(a)**	PRO 325, TRP 327, LEU 361, PHE 316, ILE 313, ARG 362, LEU467, GLN 317, GLY 321, ALA 320, ALA 323	4.51, 3.63, 4.25, 3.96, 3.79, 3.79, 4.41, 4.19, 4.05
**Standard drug acarbose**	ASP 197, ALA 198, LEU 162, HIS 305, TRP 58, LEU 165, SER 163, TRP 59, GLN 63, GLU 233, ASP 300, HIS 101, ARG 195, TYR 62, GLY 306, HIS 299, ASN 105, ALA 106, GLY 164, GLY 104, VAL 107	4.98, 5.59, 5.38, 4.66, 4.69, 6.16, 4.29, 2.61, 4.32
**Standard drug miglitol**	ASN 4, TRP 7, MET 6, PHE 463, PRO 460, ARG 457, HIS 459, ARG 456, ASP 48, LEU 462, VAL 12	5.02, 4.35, 4.87, 3.75

Surrounding amino acid residues showing interactions and possible distances with the ligands 5g, 5a & 4a(a) = 2-thion-1,3,4-oxadiazole derivatives; Å = angstrom

## Conclusion

In this study, 10 new 1,3,4-oxadiazole derivatives were synthesized and evaluated for their alpha-amylase and alpha-glucosidase inhibitory potential. These compounds are novel to the best of our knowledge and we are reporting them for the first time. Two analogues 5a and 4a(a) exhibited strong inhibitory potential against the α-glucosidase enzyme, i.e., IC_50_ value=12.27±0.41 µg/ml and 15.45±0.20 µg/ml, respectively in comparison with standard drug miglitol (IC_50_ value=11.47±0.02 µg/ml) whereas, one compound 5g demonstrated outstanding inhibitory potential (IC_50_ value=13.09±0.06 µg/ml) against the α-amylase enzyme in comparison with standard drug acarbose (IC_50_ value=12.20±0.78 µg/ml). Therefore, we suggest that 2-thione-1,3,4-oxadiazoles (5a, 5g, and 4a (a)) may act as potential lead molecules for the development of new alpha-amylase and alpha-glucosidase inhibitors.

## Authors’ Contributions

HN and MI designed the study. AB and AM conducted the experiments. AB and MI wrote the manuscript. HN and MI reviewed and edited the paper. All authors have read and approved the final version of the manuscript. 

## Conflicts of Interest

The authors declare no conflicts of interest in this study.
